# Quantitative Assessment of Forest Ecosystem Integrity and Authenticity Based on Vegetation in Hanma and Huzhong Reserves

**DOI:** 10.3390/plants15030435

**Published:** 2026-01-30

**Authors:** Xinjing Wu, Jiashuo Cao, Kun Yang, Mingliang Gao, Yongzhi Liu

**Affiliations:** 1 School of Ecology and Nature Conservation, Beijing Forestry University, Beijing 100083, China; xjing3579@163.com; 2 Inner Mongolia Greater Khingan Hanma National Nature Reserve Administration, Hulunbuir 021000, China; 3 Heilongjiang Huzhong National Nature Reserve Administration, Huzhong 165038, China; 4 Heilongjiang Lingfeng National Nature Reserve Administration, Mohe 165300, China

**Keywords:** protected area network design, forest inventory data, cold-temperate (boreal) forests, spatial completeness, ecosystem naturalness

## Abstract

Forest ecosystems provide essential ecological functions in the context of accelerating climate change. However, evaluating their conservation values and conditions remains challenging due to conceptual and methodological ambiguities. In particular, ecosystem integrity and ecosystem authenticity are often conflated in vegetation-based assessments, despite representing distinct dimensions of ecosystem condition. This study advances vegetation-based assessments by explicitly decoupling ecosystem integrity from ecosystem authenticity, while integrating spatial completeness, vegetation patterns and quality, and successional–disturbance attributes into a unified operational framework for reserve-level diagnosis and comparison. The resulting indices enable managers to distinguish boundary-driven limitations of landscape integrity from internal vegetation conditions that persist in near-natural states, thus enhancing interpretability for conservation planning in the context of climate change. Using standardized forest resource survey data and spatial analysis, we constructed two composite indices: Forest Ecosystem Integrity (FEI) and Forest Ecosystem Authenticity (FEA). These indices were applied to two adjacent cold-temperate forest nature reserves, Hanma and Huzhong, in the Greater Khingan Mountains of northeastern China, as well as to a merged spatial scenario. The results demonstrate consistently high ecosystem authenticity (>90%) across all study areas, indicating strong naturalness and successional maturity. In contrast, ecosystem integrity remains moderate (63–69%), primarily constrained by the low spatial completeness of conservation units. The spatial integration of the two reserves significantly improved ecosystem integrity without compromising authenticity, highlighting the role of boundary configuration in conservation effectiveness. By operationalizing integrity and authenticity as complementary yet distinct dimensions, this study provides a reproducible framework for evaluating forest ecosystem conditions and offers practical insights for the design of protected area networks and adaptive management in cold-temperate forest regions.

## 1. Introduction

Forest ecosystems are among the most complex terrestrial systems due to their multi-layered vegetation structure, long successional cycles, strong coupling between biotic and abiotic processes, and high sensitivity to climate and disturbance regimes [1,2]. They play a critical role in maintaining biodiversity, regulating carbon cycles, conserving water resources, and sustaining ecosystem services essential for human well-being and ecological security [3,4,5]. Under accelerating climate change and increasing anthropogenic pressures, accurately assessing forest ecosystem condition within protected areas has become a central task for conservation science and environmental governance.

In recent decades, ecosystem integrity has emerged as a core concept for evaluating protected-area condition and effectiveness. Integrity refers to the degree to which an ecosystem retains its characteristic structures, processes, and functions, as well as its capacity to sustain a balanced and self-regulating biological system within its natural range of variation [6,7,8]. This concept has been widely adopted in international conservation policy and management frameworks [5,9], including the Kunming–Montreal Global Biodiversity Framework. A wide range of integrity-based assessment frameworks have been developed and applied across multiple ecosystem types, particularly forests [10], rivers [11], wetlands [12], and composite landscapes [13,14]. In forest ecosystems, integrity assessments commonly employ indicators related to forest cover, vegetation structure, species composition and ecological processes [15,16,17].

Methodologically, ecosystem integrity assessments have evolved from qualitative expert-based evaluations toward increasingly standardized quantitative approaches [18]. Representative frameworks include the Index of Biotic Integrity (IBI [19]), the Three-Level Approach (TLA [20]), the Ecosystem Integrity Assessment Framework (EIAF [21]) and the Essential Ecosystem Characteristics (EECs) framework [16], which integrate field observations, remote sensing, and spatial modeling to capture ecosystem structure, function, and spatial organization across scales. Among practical applications, the ecological integrity assessment system developed by Parks Canada represents a widely recognized model for protected-area monitoring, providing explicit definitions, indicator systems, and management-oriented evaluation protocols [9].

Despite these advances, ecosystem integrity alone does not fully capture all dimensions of ecosystem condition, particularly in long-inhabited or historically managed forest landscapes [22]. In such contexts, ecosystems may retain intact vegetation structure and ecological processes while deviating from historical or reference-state composition due to legacy disturbances, altered successional trajectories, or past management interventions. To address this limitation, the concept of ecosystem authenticity has gained increasing attention in ecological and conservation research [23,24]. Authenticity emphasizes the extent to which current ecosystem composition, structure, and successional dynamics reflect reference or near-natural conditions, including the persistence of climatic communities, natural disturbance regimes, and minimal human modification [25].

However, compared with ecosystem integrity, authenticity remains less consistently operationalized in empirical studies. Existing research reflects divergent interpretations of authenticity: some approaches emphasize proximity to an undisturbed or “wilderness” state, relying on indicators such as human footprint, land-use intensity, remoteness from infrastructure or landscape naturalness [23,26]; others adopt a process-oriented perspective, focusing on vegetation regeneration capacity, successional trajectories, food-web complexity, and internal ecosystem dynamics [27,28]. While these approaches offer complementary insights, most authenticity assessments remain case-specific, qualitative, or weakly standardized, limiting their broader applicability and comparability across regions.

In recent protected-area research and management practice, a growing number of studies have begun to consider ecosystem integrity and authenticity in parallel [14]. In operational terms, integrity is often assessed using indicators related to area, connectivity, and landscape configuration, while authenticity is treated as a deviation-based metric relative to historical or natural benchmarks. For instance, integrity may be compromised where boundaries fail to align with ecological units, yet this does not necessarily imply internal vegetation degradation; conversely, in forests experiencing minimal disturbance and progressing along natural succession pathways, authenticity may remain remarkably high. Nevertheless, these two dimensions are frequently assessed separately, and their functional distinction remains insufficiently tested within a unified quantitative framework. Explicitly decoupling and jointly evaluating integrity and authenticity is therefore essential for accurately diagnosing conservation outcomes and identifying whether management limitations arise from boundary design, connectivity deficits, or internal ecological degradation.

The Greater Khingan Mountains, located in Northeast China, represent the largest and most intact distribution area of cold-temperate bright coniferous forest (Taiga) in the country and constitute an important component of the global boreal forest ecosystem [29]. Forest coverage in this region exceeds 95%, reflecting its exceptionally high level of ecological integrity [30]. Within this area, the Hanma National Nature Reserve in Inner Mongolia and the Huzhong National Nature Reserve in Heilongjiang Province preserve extensive tracts of contiguous, minimally disturbed old-growth forests, conferring outstanding conservation value and scientific significance. However, differences in boundary configuration, spatial coverage of natural geographic units, and disturbance history may lead to divergent integrity and authenticity outcomes, even among adjacent reserves [22,31]. A systematic assessment that explicitly separates these two dimensions is therefore needed to support adaptive conservation strategies and protected-area network optimization.

Against this background, this study develops and applies a vegetation-based framework to quantitatively assess forest ecosystem integrity and authenticity in Hanma and Huzhong National Nature Reserves. Specifically, we aim to:

(1) Construct an indicator system that explicitly separates ecosystem integrity and authenticity. Ecosystem integrity is characterized by spatial completeness, vegetation pattern, and vegetation quality, while ecosystem authenticity is represented by climatic community representation, successional authenticity, and forest quality authenticity;
(2) Quantify and map comprehensive indices of forest ecosystem integrity (FEI) and authenticity (FEA) for each reserve and for a merged region scenario;
(3) Evaluate how boundary configuration and reserve integration influence integrity and authenticity, with implications for protected-area boundary optimization and network design under climate change.

By decoupling integrity and authenticity and testing a merged-boundary scenario, this study provides a diagnostic framework that distinguishes boundary-driven landscape constraints from internal vegetation reference-state consistency, offering practical insights for forest conservation assessment and policy-oriented decision making.

## 2. Materials and Methods

### 2.1. Study Area

**Figure 1:**
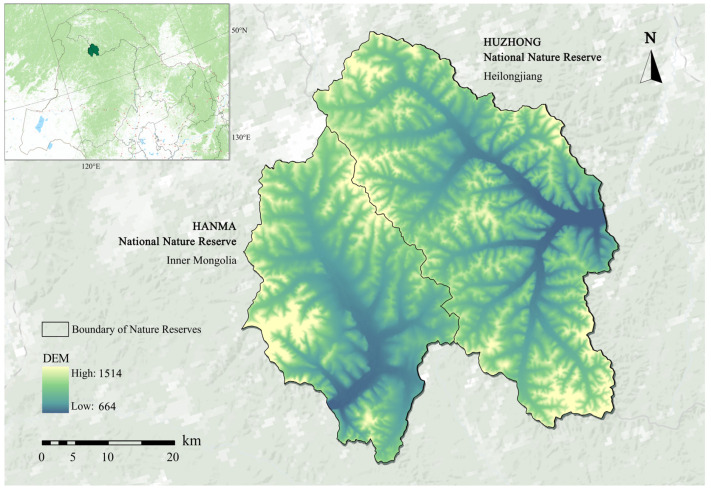
Location map of the study areas. DEM: Digital Elevation Model.

This study was conducted in two adjacent national nature reserves located in the Greater Khingan Mountains of northeastern China, representing typical cold-temperate coniferous forest ecosystems. The Hanma National Nature Reserve is situated on the northern part of the western slope of the Greater Khingan Mountains in Inner Mongolia Autonomous Region (approximately 122°23′–122°53′ E, 51°20′–51°50′ N). The Huzhong National Nature Reserve is located on the northeastern slope of the Greater Khingan Mountains in Heilongjiang Province (approximately 122°42′–123°18′ E, 51°18′–51°56′ N) [32]. The two reserves are separated by the main ridge of the Greater Khingan Mountains and together encompass one of the largest and most representative tracts of cold-temperate forest remaining in China (Figure 1). The Digital Elevation Model (DEM) used in Figure 1 was derived from the Copernicus Digital Elevation Model (COP-DEM), provided by the European Space Agency (ESA).

The regional climate is characterized by a cold-temperate continental monsoon regime, with long, cold winters and short, cool summers. Mean annual temperatures are generally below 0 °C, and precipitation is concentrated during the growing season. These climatic conditions, combined with high latitude and elevation, strongly constrain forest growth and succession, shaping distinctive vegetation structure and composition across the landscape.

Forest vegetation dominates both reserves and constitutes the primary conservation target. The zonal vegetation is classified as cold-temperate bright coniferous forest, with Dahurian larch (*Larix gmelinii*) as the dominant tree species. Along altitudinal gradients, vegetation exhibits a clear vertical zonation pattern, including subalpine dwarf forest dominated by Siberian dwarf pine (*Pinus pumila*), cold-temperate coniferous sparse forest, and cold-temperate coniferous forest at lower elevations [32,33,34,35]. These forest communities represent typical climatic and topographic climax assemblages of the Greater Khingan Mountains.

Both reserves retain large areas of relatively undisturbed natural forest and display limited human disturbance due to long-term protection. At the same time, spatial fragmentation [36], historical fire events [37], and differences in reserve boundaries [38] result in heterogeneity in vegetation patterns and forest structure. This combination of high naturalness and spatial variability makes the Hanma and Huzhong National Nature Reserves well suited for assessing forest ecosystem integrity and authenticity and for exploring the effects of spatial integration of adjacent protected areas.

### 2.2. Data Sources

Multiple data sources were integrated to support the assessment of forest ecosystem integrity and authenticity. These included forest resource survey data, spatial and geographic datasets, as well as supplementary investigation and planning materials. Collectively, these datasets provided essential structural, compositional, spatial, and management-related information necessary for a comprehensive evaluation of forest ecosystem conditions in the study areas.

#### 2.2.1. Forest Resource Survey Data

The primary data source for this study was the forest resource survey data. Specifically, data from the Forest Resources Second-Class Inventory of the Hanma National Nature Reserve and Huzhong National Nature Reserve (survey year: 2016) were utilized. In China, the Forest Resources Second-Class Inventory, also referred to as the forest management planning and design inventory, is a standardized national forest survey conducted at the forest sub-compartment level. This inventory is extensively employed in forest management and resource assessment.

The dataset offers comprehensive information on forest stand structure and site conditions, including forest type, stand origin, age group, canopy density, diameter at breast height, canopy height, site quality, soil thickness, and disturbance attributes. These data were obtained from the local forestry authorities responsible for reserve management and were accessed in 2022 for research purposes.

#### 2.2.2. Spatial and Geographic Data

Spatial and geographic datasets were utilized to delineate reserve boundaries, characterize vegetation distribution, and analyze landscape structure. The comprehensive geographical regionalization dataset for natural conservation in China was employed to identify fourth-level natural conservation geographical units [39] and to calculate indicators related to the integrity of conservation zones.

Land-use spatial data were derived from official reserve planning documents and scientific investigation reports. These datasets facilitated the distinction between protective and non-protective vegetation types and supported the calculation of indicators for vegetation pattern integrity and authenticity. All spatial datasets were projected into a unified coordinate reference system and processed to ensure spatial consistency and comparability.

#### 2.2.3. Supplementary Investigation and Planning Materials

Supplementary data sources included official scientific investigation reports, overall management planning documents, and other relevant archival materials from both nature reserves. These documents, provided by the reserve management authorities, contain baseline information on vegetation classification, community types, and conservation and management objectives, serving as reference materials to support data interpretation.

Additionally, field investigations were conducted from 2022 to 2025 as part of this study to verify vegetation types, identify characteristics of climax communities, and assess disturbance conditions in selected forest sub-compartments. The field surveys focused on confirming land-use types and vegetation classifications derived from existing datasets, as well as documenting observable signs of natural or anthropogenic disturbances. The results of these field investigations supplemented and refined the forest resource survey data and spatial datasets, thereby enhancing the accuracy and reliability of the assessment of forest ecosystem integrity and authenticity.

Collectively, these supplementary investigation and planning materials provided essential on-site verification and contextual support for the integrated analysis of forest ecosystem conditions.

### 2.3. Indicator System and Calculation

Based on the principles of scientific validity, ecological relevance, representativeness, and operational feasibility [40], a vegetation-based indicator system has been constructed to quantitatively assess the integrity and authenticity of forest ecosystems. Vegetation was selected as the primary evaluation object because it encapsulates long-term climatic constraints, disturbance history, and ecosystem processes, thereby providing a direct reflection of ecosystem structure, function, and successional status [41,42].

The indicator system comprises two complementary dimensions: forest ecosystem integrity and forest ecosystem authenticity. Integrity indicators focus on spatial completeness, structural continuity, and the overall quality of forest ecosystems, while authenticity indicators emphasize naturalness, successional maturity, and the degree of anthropogenic disturbance in relation to reference conditions. The structure of the indicator system is summarized in Table 1.

#### 2.3.1. Forest Ecosystem Integrity

The integrity of forest ecosystems was assessed using three indicators that capture spatial, structural, and quality-related attributes.

1. Conservation Zone Integrity (CZI)

Conservation Zone Integrity reflects the degree to which the spatial boundaries of a protected area align with complete natural protection geographical units. This indicator was quantified by analyzing the proportion of the study area situated within fourth-level natural conservation geographical units [39], thus evaluating whether reserve boundaries encompass relatively intact natural units rather than fragmented sections. Higher CZI values signify greater spatial completeness and a stronger capacity to sustain ecosystem processes at the landscape scale.

**Figure 2:**
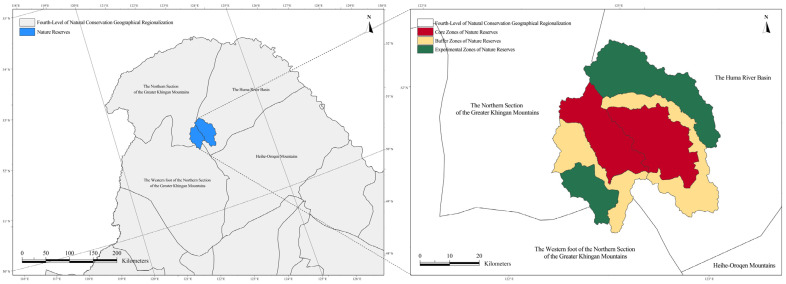
Location of the Hanma and Huzhong National Nature Reserves in relation to the fourth-level natural conservation geographical subregions.

From the perspective of the fourth-level units of natural conservation geographical regionalization, the Hanma National Nature Reserve is predominantly situated within the northern section of the Greater Khingan Mountains, which encompasses approximately 68.05% of the reserve’s total area. The remaining portions are distributed across the Huma River Basin and the western foot of the northern section of the Greater Khingan Mountains. In contrast, the Huzhong National Nature Reserve displays a more spatially concentrated distribution, with approximately 89.84% of its area located within the Huma River Basin, while only a small fraction extends into the western foot of the northern section of the Greater Khingan Mountains. The spatial relationships between the two reserves and the fourth-level natural conservation geographical units are depicted in Figure 2.

2. Vegetation Pattern Integrity (VPI)

Vegetation Pattern Integrity characterizes the spatial configuration and connectivity of protective vegetation within a protected area. Protective vegetation is defined as forest land that directly contributes to ecosystem functioning, whereas non-protective vegetation includes non-forest land, burned land, and other land-cover types with limited ecological protection value [2]. VPI integrates the proportion of protective vegetation, landscape fragmentation, and edge effects to reflect the continuity and structural coherence of vegetation mosaics. The integrity of protective vegetation within the nature reserve is analyzed based on the area of the protective vegetation mosaic, fragmentation indicator ($I_{F}$), edge effect ($I_{E}$), and other relevant metrics. The fragmentation indicator ($I_{F}$) and edge effect ($I_{E}$) reflect the overall degree of fragmentation of all protective vegetation. This indicator captures the influence of spatial patterns on ecosystem integrity and resilience.

3. Vegetation Quality Integrity (VQI)

Vegetation Quality Integrity evaluates the overall quality of forest stands based on structural and developmental attributes derived from forest resource survey data. Indicators such as stand origin, age group, canopy density, mean diameter at breast height, and canopy height are employed to represent the forest’s growth status and structural condition. Higher VQI values indicate better forest stand quality and a stronger support for ecosystem functions.

Detailed calculation procedures for the Canopy Zonation Index (CZI), Vegetation Productivity Index (VPI), and VQI, including mathematical formulations and parameter definitions, are provided in Supplementary Materials S1.

#### 2.3.2. Forest Ecosystem Authenticity

Forest ecosystem authenticity was assessed using three indicators designed to reflect naturalness, successional continuity, and disturbance intensity.

1. Vegetation Pattern Authenticity (VPA)

Vegetation Pattern Authenticity refers to the proportion of natural vegetation relative to the total vegetated area within a protected area. Analyzing the degree of naturalness of forest vegetation, or the gap between its current state and its natural condition, is fundamental to evaluating forest naturalness. The evaluation process aims to understand the differences between the current status of forest vegetation in a specific area and its original (or potential) state [45]. This involves quantitative classification and assessment. The extent of natural vegetation serves as an indicator of the forest’s naturalness and further reflects the authenticity of the forest ecosystem.

2. Community Succession Authenticity (CSA)

Community Succession Authenticity quantifies the proportion of climax or near-climax plant communities within a protected area. A climax community is defined as a stable ecological community where succession concludes, allowing populations to persist through reproduction. In such communities, environmental factors are balanced, and constituent species maintain equilibrium within their habitat [46]. The size of the plant community at the climax successional stage serves as an indicator of the ecosystem’s successional status, reflecting its authenticity. Climax plant communities are characterized by long-term stability under consistent regional climate and site conditions [47], including temperature and precipitation regimes, soil moisture and nutrient availability, and topographic settings, particularly in the absence of major disturbances such as fire, windthrow, flooding, or intensive human land use. Climax communities are identified based on regional vegetation classifications and ecological reference conditions [48]. A higher CSA value indicates that a larger proportion of vegetation has reached advanced successional stages, reflecting long-term ecosystem stability and limited disturbance.

3. Forest Quality Authenticity (FQA)

Forest naturalness serves as an indicator reflecting the extent of external interference experienced by a forest [49]. A forest that experiences minimal disturbance from external sources, particularly anthropogenic pressures such as nearby infrastructure, roads, agricultural land use and conversion, logging, and associated tree-cover loss, as well as landscape fragmentation, exhibits a high level of naturalness. Conversely, a forest subjected to significant external interference demonstrates a lower level of naturalness. The lesser the degree of disturbance, the closer the forest remains to its original state [50]. This indicator was derived from attributes of forest resource surveys, including stand origin, age group, soil depth, interference intensity, and growth condition [44]. Lower disturbance levels and more natural stand characteristics correspond to higher FQA values.

Detailed calculation methods and grading schemes for authenticity indicators are presented in Supplementary Materials S1.

#### 2.3.3. Composite Indicators and Weighting Scheme

To synthesize individual indicators into overall measures of ecosystem condition, two composite indices were constructed: Forest Ecosystem Integrity index (FEI) and Forest Ecosystem Authenticity index (FEA). Each composite index was calculated as a weighted aggregation of its corresponding indicators.

Indicator weights were determined using an expert judgment approach. A panel of experts familiar with forest ecology, conservation planning, and the study region independently assigned weights to each indicator based on ecological importance, relevance to conservation objectives, and data reliability. Final weights were obtained by averaging expert scores. The resulting weighting scheme is presented in Table 2. To enhance transparency and reproducibility, all composite index calculations were implemented using standardized workflows implemented in ArcGIS Pro (version 3.0.0). Mathematical expressions for composite indices and weighting procedures are provided in Supplementary Materials S1.

### 2.4. Data Processing and Analysis Workflow

**Figure 3:**
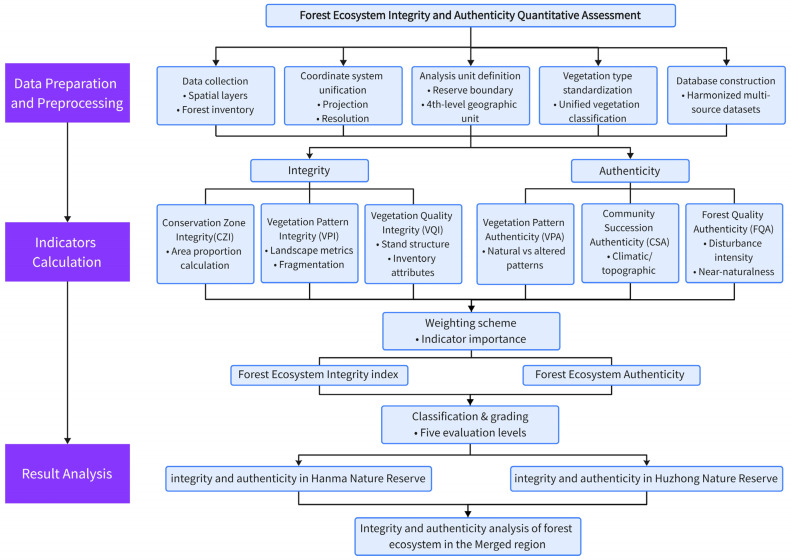
Data processing and analysis workflow for assessing forest ecosystem integrity and authenticity.

Data processing and analysis adhered to a structured workflow aimed at quantifying the integrity and authenticity of forest ecosystems (Figure 3). Initially, multi-source spatial data and forest inventory data were harmonized by unifying coordinate systems, spatial resolutions, and analysis units. The boundaries of reserves were overlaid with fourth-level natural geographic units to facilitate an analysis of spatial completeness, while vegetation types were standardized across reserves to ensure comparability.

Ecosystem integrity was assessed using indicators that described spatial completeness, vegetation patterns, and vegetation quality, derived from area-based calculations, landscape metrics, and forest inventory attributes, respectively. Concurrently, ecosystem authenticity was evaluated through indicators reflecting the authenticity of vegetation patterns, community succession, and forest quality, capturing aspects of naturalness, successional representation, and disturbance intensity.

All indicators were normalized and integrated using a predefined weighting scheme to generate composite indices of forest ecosystem integrity and authenticity, which were subsequently classified into five evaluation levels. Spatial analyses were conducted for Hanma Nature Reserve, Huzhong Nature Reserve, and their merged region to facilitate comparative assessments and diagnostic interpretations.

### 2.5. Classification and Grading

The purpose of grading the evaluation results is to qualitatively interpret whether and to what extent forest protection and management goals have been achieved [16]. By translating quantitative evaluation scores into clearly defined grades, this grading approach provides an intuitive and policy-relevant summary of assessment outcomes. It enables forest managers, policymakers, and the general public to track progress toward stated forest conservation and management objectives and facilitates consistent comparison with future conservation outcomes.

For the evaluation indicators in Table 1, based on relevant research [27], the evaluation results are classified into five levels. The grades are categorized as follows:

The evaluation value is [80%, 100%]: The evaluation level is “good”, indicating a relatively complete ecosystem structure and well-functioning ecological processes. Forests categorized in this manner typically demonstrate robust recovery and resilience capabilities, devoid of significant ecological issues commonly linked to forest degradation. Such issues include intense anthropogenic disturbances, habitat fragmentation, structural simplification, biodiversity loss, and degradation of soil and site conditions.

The evaluation value is [60%, 80%): The evaluation level is “better”, which means that the ecosystem structure is relatively complete, the functions are good, it can be restored under normal disturbance, and there are no major ecological problems.

The evaluation value is [50%, 60%): The evaluation level is “average”, which means that the structure of the ecosystem is changing, but the basic functions can still be maintained. After being disturbed, the ecosystem is likely to deteriorate and ecological problems will occur.

The evaluation value is [30%, 50%): The evaluation level is “worse”, which means that the ecosystem structure is greatly damaged, the functions are degraded and incomplete, it is difficult to recover after being disturbed by the outside world, and the ecological problems are serious.

The evaluation value is [0, 30%): the evaluation level is “bad”, which means that the ecosystem structure is incomplete, functions are lost, it is difficult to restore and rebuild the ecosystem, and the ecological problems are severe.

## 3. Results

### 3.1. Overall Patterns of Forest Ecosystem Integrity and Authenticity

**Figure 4:**
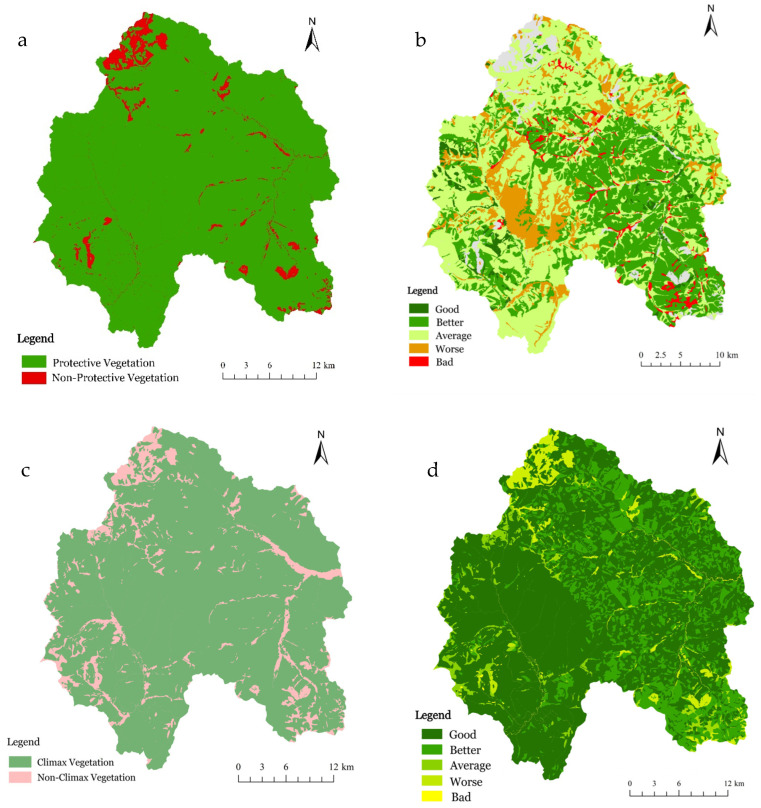
Spatial distributions of vegetation-based integrity and authenticity indicators. (**a**) Mosaic distribution of protected and unprotected vegetation used to derive Vegetation Pattern Integrity (VPI); (**b**) Spatial distribution of Vegetation Quality Integrity (VQI) classified into five levels; (**c**) Mosaic distribution of climax and non-climax vegetation representing Community Succession Authenticity (CSA); (**d**) Spatial distribution of Forest Quality Authenticity (FQA) classified into five levels.

Across all evaluated spatial units, including the Hanma National Nature Reserve, the Huzhong National Nature Reserve, and the merged region, a clear and consistent divergence was observed between ecosystem integrity and ecosystem authenticity (Table 3 and Figure 4). Figure 4 illustrates the spatial distributions of key integrity and authenticity indicators across the study area. Specifically, Figure 4a depicts the mosaic pattern of protected and unprotected vegetation used to characterize Vegetation Pattern Integrity (VPI), where protected vegetation predominates in most areas, while patches of unprotected vegetation are predominantly found along edges and localized zones. Figure 4b presents the spatial distribution of Vegetation Quality Integrity (VQI), classified into five levels ranging from good to poor. High and moderate VQI values are widely distributed throughout the reserve, whereas lower values are observed in relatively fragmented or structurally simplified forest patches. The spatial pattern of Community Succession Authenticity (CSA) is illustrated in Figure 4c, distinguishing climax vegetation from non-climax vegetation. Climax vegetation constitutes the majority of the reserve area, indicating that forest communities are largely in advanced successional stages. Figure 4d displays the distribution of Forest Quality Authenticity (FQA), which reflects disturbance intensity and near-natural stand conditions. Most forest stands exhibit good to excellent FQA levels, with only limited areas showing lower authenticity. Collectively, these spatial patterns demonstrate that, while internal vegetation conditions and successional status remain largely intact, there are variations in attributes related to integrity and authenticity at fine spatial scales across the reserve. Overall, ecosystem authenticity remains uniformly high, whereas ecosystem integrity is moderate, revealing contrasting dimensions of ecosystem condition.

In terms of composite indices, Forest Ecosystem Authenticity (FEA) exceeded 90% across all three spatial units, indicating a high degree of naturalness, advanced successional status, and limited anthropogenic disturbance. In contrast, Forest Ecosystem Integrity (FEI) ranged from 62.91% to 69.34%, corresponding to a ‘Better’ evaluation level. This discrepancy suggests that while the forests maintain strong historical and ecological authenticity, their integrity at the landscape and spatial-structural levels is comparatively constrained.

The analysis of individual integrity indicators provides further clarification of this pattern. Vegetation Pattern Integrity (VPI) consistently exhibited high values (≥80%) across all study areas, indicating relatively continuous and well-connected protective vegetation mosaics within the boundaries of the reserves. In contrast, Vegetation Quality Integrity (VQI) demonstrated intermediate values (approximately 54–57%), which suggests heterogeneity in forest stand structure and quality. This variation is likely associated with differences in age structure, canopy characteristics, and site conditions. Conversely, Conservation Zone Integrity (CZI) was uniformly low in all cases (<5%), underscoring that the spatial boundaries of the reserves encompassed only a small proportion of complete fourth-level natural protection geographical units, which constitutes the primary constraint on overall ecosystem integrity.

For ecosystem authenticity, all three component indicators exhibited consistently high values. Vegetation Pattern Authenticity (VPA) reached 100% across all study areas, indicating that the vegetation coverage within the reserves is entirely dominated by natural vegetation types. Community Succession Authenticity (CSA) exceeded 90%, reflecting a substantial proportion of climax or near-climax plant communities and long-term successional stability. Forest Quality Authenticity (FQA) also remained elevated (>85%), suggesting generally low levels of external disturbance and a strong persistence of natural forest characteristics at the stand level.

Taken together, these results reveal a systematic contrast between high ecosystem authenticity and moderate ecosystem integrity across the study areas. While the forests exhibit strong naturalness and successional maturity, their spatial completeness and landscape-scale structural integrity remain limited, as evidenced by low conservation zone integrity. This overall pattern underscores the analytical value of treating ecosystem integrity and authenticity as distinct yet complementary dimensions, thereby enabling a more nuanced and diagnostically meaningful assessment of forest ecosystem conditions within protected areas.

### 3.2. Comparison Between Hanma and Huzhong Nature Reserves

Although the Hanma and Huzhong National Nature Reserves are geographically adjacent and share similar cold-temperate coniferous forest types, notable differences in specific indicators of ecosystem integrity and authenticity were observed (Table 3). Overall, the Forest Ecosystem Integrity (FEI) of the Huzhong Nature Reserve (63.49%) was slightly higher than that of the Hanma Nature Reserve (62.91%). In contrast, the Forest Ecosystem Authenticity (FEA) values were comparably high in both reserves, reaching 92.82% and 91.59%, respectively.

The differences between the two reserves were primarily associated with integrity-related indicators. Both reserves exhibited very low Conservation Zone Integrity (CZI), reflecting limited spatial coverage relative to complete fourth-level natural protection geographical units. However, the Huzhong Nature Reserve demonstrated a higher CZI value of 3.32% compared to the Hanma Nature Reserve’s 1.93%, indicating a slightly greater degree of spatial completeness. While Vegetation Pattern Integrity (VPI) was high in both reserves, the internal structure varied: Hanma exhibited a higher fragmentation indicator, whereas Huzhong displayed a stronger edge effect. This suggests notable differences in the configuration and shape complexity of the protective vegetation mosaics.

The Vegetation Quality Integrity (VQI) also varied between the two reserves. The VQI value for the Huzhong Nature Reserve (56.89%) was slightly higher than that of the Hanma Nature Reserve (54.07%), indicating a relatively superior overall quality of the forest stands. This discrepancy reflects variations in the composition of forest age, canopy density, and structural attributes, as documented by the forest resource survey data.

In contrast, the indicators of ecosystem authenticity were consistently high and exhibited limited variation between the two reserves. The Vegetation Pattern Authenticity (VPA) reached 100% in both instances, indicating that vegetation coverage is entirely dominated by natural vegetation types. The Community Succession Authenticity (CSA) exceeded 90% in both reserves, with Huzhong demonstrating a slightly higher value (95.07%) compared to Hanma (90.79%). The Forest Quality Authenticity (FQA) values were also comparable, recorded at 85.74% for Huzhong and 85.87% for Hanma.

Overall, the comparison indicates that while the two reserves differ moderately in terms of spatial and structural aspects of ecosystem integrity, they exhibit similarly high levels of ecosystem authenticity. These results suggest that differences between the reserves are more pronounced in landscape-scale integrity than in naturalness or successional status. This pattern reinforces the distinction between integrity and authenticity as complementary but non-equivalent dimensions of forest ecosystem condition.

### 3.3. Effects of Spatial Integration on Ecosystem Integrity and Authenticity

The spatial integration of the Hanma and Huzhong National Nature Reserves into a unified assessment unit has led to a significant enhancement of forest ecosystem integrity while preserving a high degree of ecosystem authenticity (Table 3). The Forest Ecosystem Integrity (FEI) of the merged region increased to 69.34%, surpassing the values recorded in both individual reserves. In contrast, the Forest Ecosystem Authenticity (FEA) remained consistently high at 92.31%, comparable to the levels documented in the Hanma and Huzhong reserves.

The improvement in ecosystem integrity following spatial integration was primarily driven by changes in the characteristics of vegetation patterns. The Vegetation Pattern Integrity (VPI) increased significantly to 93.11% in the merged region, indicating reduced fragmentation and a diminished edge effect of protective vegetation mosaics compared to the individual reserves. This suggests that spatial integration enhanced landscape connectivity and the structural continuity of protective vegetation across the combined area.

The Conservation Zone Integrity (CZI) increased to 4.84% following integration. Although this value remains low in absolute terms, it signifies a notable improvement compared to the individual reserves. This suggests that spatial integration enhances the proportion of complete natural protection geographical units within the protected area. The Vegetation Quality Integrity (VQI) in the merged region remained at an intermediate level of 55.75%, which is comparable to the values observed in the individual reserves. This indicates that spatial integration primarily affects landscape configuration rather than stand-level vegetation quality.

Ecosystem authenticity indicators exhibited limited change following spatial integration. Vegetation Pattern Authenticity (VPA) remained at 100%, while Community Succession Authenticity (CSA) and Forest Quality Authenticity (FQA) consistently maintained high levels at 93.30% and 85.79%, respectively. These results indicate that spatial integration does not alter the naturalness or successional characteristics of forest ecosystems; rather, it enhances their integrity by improving spatial coherence and reducing fragmentation.

The merged-region analysis demonstrates that the spatial integration of adjacent protected areas can significantly enhance forest ecosystem integrity without compromising ecosystem authenticity. This pattern underscores the importance of spatial configuration and boundary design in influencing ecosystem integrity outcomes at the landscape scale.

## 4. Discussion

### 4.1. Implications of Separating Ecosystem Integrity and Authenticity

A central objective of this study was to examine whether forest ecosystem integrity and ecosystem authenticity can be meaningfully distinguished in quantitative assessments based on vegetation characteristics. The results clearly demonstrate that these two dimensions do not converge in cold–temperate forest ecosystems, as consistently high authenticity values contrast with only moderate levels of integrity across all evaluated spatial units.

This finding is broadly consistent with earlier studies reporting high naturalness and advanced successional stages in the forests of the Greater Khingan Mountains [51,52]. Previous assessments based on forest age structure, species composition, or disturbance intensity have similarly concluded that these forests remain close to their natural or historical states. The high values of Vegetation Pattern Authenticity, Community Succession Authenticity, and Forest Quality Authenticity observed in this study align with these conclusions, confirming that the vegetation-based indicators used here reliably capture long-term ecological continuity.

However, this study departs from much of the existing literature by demonstrating that high authenticity does not necessarily imply high ecosystem integrity. Unlike many previous evaluations that implicitly treat naturalness as a proxy for integrity, the present results reveal that integrity is strongly constrained by spatial completeness and landscape configuration. This divergence arises because ecosystem integrity, as operationalized here, explicitly incorporates spatial correspondence between protected areas and natural geographical units [39], a dimension that is rarely addressed in earlier vegetation-focused assessments. The separation of ecosystem integrity and authenticity allows for a more precise diagnosis of conservation status by providing a spatially explicit diagnostic framework for identifying structural constraints on conservation effectiveness, thereby avoiding the underestimation of ecosystem threat status based solely on internal vegetation characteristics.

### 4.2. Landscape Configuration and Spatial Constrains on Ecosystem Integrity

The comparative analysis between the Hanma and Huzhong Nature Reserves provides valuable insights into how spatial factors influence ecosystem integrity. Although the two reserves share similar climatic conditions, forest types, and protection histories, their integrity indicators differ in terms of fragmentation, edge effects, and spatial completeness. These differences are primarily reflected in Vegetation Pattern Integrity and Conservation Zone Integrity, while authenticity indicators remain consistently high.

This pattern aligns with landscape-ecological studies that emphasize the significance of connectivity, patch size, and boundary effects in maintaining ecosystem processes [53]. Previous research has indicated that fragmented protected areas may preserve high-quality habitats internally while still facing limitations in landscape-scale ecological functions [54]. The present results corroborate this perspective by demonstrating that spatial constraints can restrict ecosystem integrity, even in regions with minimal direct human disturbance. It is important to note that Conservation Zone Integrity (CZI) is intentionally conservative, as it assesses the proportional coverage of complete natural geographic units rather than the internal condition of ecosystems. Consequently, low CZI values do not indicate methodological bias; they reflect the stringent spatial criteria adopted in this study. Importantly, these spatial limitations should not be interpreted as evidence of management failure, but rather as outcomes of reserve delineation concerning natural geographic units, underscoring structural constraints imposed by boundary configurations rather than deficiencies in conservation practices [55].

The moderate Vegetation Quality Integrity values observed in both reserves should be interpreted in the context of regional ecological conditions. Cold-temperate forests are characterized by slow growth and relatively low canopy stature, largely because mean annual temperatures are typically below 0 °C (approximately −4 to −6 °C in the study reserves) and the growing season is short, generally spanning April–September [56]. Cold-temperate forests are characterized by slow growth rates, small tree stature, and a relatively simple vertical structure due to low temperatures and short growing seasons [57]. Similar structural patterns have been reported in previous studies of high-latitude forest ecosystems and are not necessarily indicative of degradation [58]. The consistency between these findings and earlier research suggests that the vegetation quality indicators employed in this study are sensitive to ecological context rather than artificially inflating or deflating integrity assessments.

### 4.3. Effects of Spatial Integration on Integrity and Authenticity

The merged-region analysis directly addresses whether the spatial integration of adjacent protected areas can improve ecosystem condition, a question of practical relevance for conservation planning. The results indicate that the integration of the Hanma and Huzhong Nature Reserves substantially enhances ecosystem integrity while largely preserving ecosystem authenticity.

This outcome aligns with studies demonstrating that larger and more contiguous protected areas tend to support higher landscape-level integrity by reducing fragmentation and edge effects [53]. The significant increase in Vegetation Pattern Integrity and the improvement in Conservation Zone Integrity following spatial integration suggest that the proposed framework effectively captures these spatial benefits. In contrast, the stability of authenticity indicators implies that integration does not alter the internal ecological character of forests, which remains governed by long-term successional processes and disturbance history.

The combination of these results highlights an important distinction: spatial integration primarily enhances the structural and spatial dimensions of ecosystem integrity, rather than modifying vegetation composition or successional status. This finding elucidates why authenticity remains high both before and after integration and underscores the complementary roles of internal ecosystem condition and spatial configuration in determining overall conservation effectiveness. In this regard, spatial integration not only improves current ecosystem integrity but also enhances the capacity of protected areas to accommodate future climate-driven disturbances [59], providing empirical support for the delineation of large-scale national parks or interconnected national park networks and for adaptive conservation planning in cold-temperate forest regions [60].

### 4.4. Methodological Considerations and Scope of Inference

The results of this study demonstrate that a vegetation-based framework can effectively address key scientific questions related to forest ecosystem integrity and authenticity, particularly in regions with long-term field investigations and well-established forest resource survey datasets. By explicitly separating these two dimensions, the framework provides a more nuanced interpretation of ecosystem conditions than approaches that rely on a single composite index. However, the extent to which the framework addresses broader questions regarding ecosystem functioning and resilience remains limited. While vegetation indicators capture structural, compositional, and successional attributes, functional processes such as nutrient cycling, fire dynamics, and species interactions are represented indirectly. This limitation aligns with previous critiques of vegetation-based assessments and suggests that future studies should integrate complementary data sources, such as long-term monitoring or remote sensing time series, to more effectively capture dynamic ecosystem processes.

Overall, these results suggest that the framework is particularly well suited for diagnosing spatial and structural constraints on conservation effectiveness in forest ecosystems, rather than serving as a comprehensive proxy for all aspects of ecosystem functioning.

## 5. Conclusions

This study develops a vegetation-based framework that explicitly separates ecosystem integrity from ecosystem authenticity, providing a diagnostically clearer approach to evaluating protected-area effectiveness. When applied to two adjacent cold-temperate reserves, the framework reveals a consistent divergence between the two dimensions: ecosystem authenticity remains uniformly high (>90%), reflecting near-natural composition, successional maturity, and minimal human disturbance. In contrast, ecosystem integrity is moderate (63–69%), primarily constrained by the low spatial completeness of conservation geographical units, a limitation more linked to boundary design than to internal ecological degradation. The spatial integration of the reserves further clarifies this distinction: merging them significantly improves landscape-level integrity, mainly through enhanced vegetation-pattern connectivity and greater coverage of complete geographical units, without reducing their high authenticity. These findings offer a transferable, indicator-based method for diagnosing whether conservation limitations stem from spatial-configuration constraints or deviations from reference ecological states. For policymakers and managers, this supports more targeted interventions such as boundary adjustment, connectivity enhancement, and climate-adaptive network planning. Future studies should test the framework across more forest types and governance contexts, incorporating temporal data and independent disturbance records to further validate its diagnostic robustness and generalizability.

## Figures and Tables

**Table 1:** Methodological framework of integrity and authenticity of forest ecosystems.

Evaluation Content	Serial Number	Evaluating Indicator	Symbol	Indicator Meaning
Forest Ecosystem Integrity (FEI)	1	Conservation Zone Integrity	CZI	The maximum value of the proportion of the study area in each fourth-level natural protection comprehensive geographical division unit to the total area of the fourth-level unit [27].
	2	Vegetation Pattern Integrity	VPI	The overall integrity of the protective vegetation is comprehensively reflected by the area proportion, fragmentation indicator and edge effect indicator of the protective vegetation mosaic [27].
	3	Vegetation Quality Integrity	VQI	Data from forest survey data are used as indicators to comprehensively reflect the overall quality of forest vegetation in the protected area [43].
	Forest Ecosystem Integrity		FEI	Comprehensive assessment of forest ecosystem integrity [27].
Forest Ecosystem Authenticity (FEA)	1	Vegetation Pattern Authenticity	VPA	The area of natural vegetation accounts for the proportion of the total area covered by vegetation in the protected area [27].
	2	Community Succession Authenticity	CSA	The proportion of the area of top plant communities to the total area of vegetation coverage in the protected area [27].
	3	Forest Quality Authenticity	FQA	Data from forest survey data are used as indicators to comprehensively reflect the degree of forest disturbance within the protected area [44].
	Forest Ecosystem Authenticity		FEA	Comprehensive evaluation of forest ecosystem authenticity [27].

**Table 2:** Indicator assignment for forest ecosystem integrity and authenticity.

Evaluation Indicators		Indicator Assignment
Forest ecosystem integrity	Conservation Zone Integrity	0.1
	Vegetation Pattern Integrity	0.5
	Vegetation Quality Integrity	0.4
Forest ecosystem authenticity	Vegetation Pattern Authenticity	0.3
	Community Succession Authenticity	0.3
	Forest Quality Authenticity	0.4

**Table 3:** Comparison of forest ecosystem integrity and authenticity indicators across the study areas.

Evaluation Content	Symptom	Hanma NR	Huzhong NR	Merged Region	Evaluation Level
Forest ecosystem integrity	CZI (%)	1.93	3.32	4.84	Bad
	VPI (%)	82.28	80.80	93.11	Good
	VQI (%)	54.07	56.89	55.75	Average
	FEI (%)	62.91	63.49	69.34	Better
Forest ecosystem authenticity	VPA (%)	100.00	100.00	100.00	Good
	CSA (%)	90.79	95.07	93.30	Good
	FQA (%)	85.87	85.74	85.79	Good
	FEA (%)	91.59	92.82	92.31	Good

## Data Availability

The original contributions presented in this study are included in the article/Supplementary Material. Further inquiries can be directed to the corresponding author.

## Supplementary Materials

The following supporting information can be downloaded at: https://www.mdpi.com/article/10.3390/plants15030435/s1, S1. Mathematical formulations and parameter definitions of ecosystem integrity and authenticity indicators; Table S1. Grading of indicators for the overall forest vegetation quality indicator; Table S2. Grading of indicators for the Forest Quality Authenticity.
