# Supplementary material for: Quantitative Assessment of Forest Ecosystem Integrity and Authenticity Based on Vegetation in Hanma and Huzhong Reserves
Source: Plants (Basel). 2026 Jan 30;15(3):435. doi: 10.3390/plants15030435 (PMC12899305; doi:10.3390/plants15030435)
Supplement: Supplementary file 1 [file plants-15-00435-s001.zip › plants-4075075-supplementary.pdf]

S1. Mathematical formulations and parameter definitions of ecosystem integrity and authenticity indicators

S1.1 Forest Ecosystem integrity

(1) Conservation Zone Integrity (CZI) [1]

$$\mathrm{CZI} = \max(S_i/S_{Gi}) \times 100\% \tag{S1}$$

Where：

CZI— Protected Area Integrity Indicator, taking the maximum value of the coverage ratio of the study area in each four-level natural protection comprehensive geographical division unit, ranging from 0 to 100%; when the study area covers a complete four-level geographical unit, CZI=100%；

S_i— The area occupied by the study area in the i-th fourth-level geographical unit;

S_{Gi}— The area in the i-th fourth-level geographical unit;

100%—Conversion constant , convert the value range of CZI to between 0 and 100%.

(2) Vegetation Pattern Integrity (VPI) [1]

$$\mathrm{VPI}=\sqrt[3]{\sum_{i=1}^{n} S_i \Big/ S_N \times (1-I_F) \times (2-I_E)} \times 100\% \tag{S2}$$

$$\mathrm{IF}=1-\sum_{i=1}^{n}\left(\frac{S_i}{\sum_{i=1}^{n} S_i}\right)^2 \tag{S3}$$

$$I_E=\sum_{i=1}^{n}\left[\frac{S_i}{\sum_{i=1}^{n} S_i} \times \frac{2\lg(0.25P_i)}{\lg S_i}\right] \tag{S4}$$

Where:

VPI— Protective vegetation integrity indicator, ranging from 0 to 100%;

S_i—The area of the i-th protective vegetation mosaic;

S_N—The total area of the study area;

n—The number of protective vegetation mosaics;

I_F—Protective vegetation fragmentation indicator, which reflects the overall fragmentation degree of protective vegetation within the study area, ranging from 0 to 1.

I_E—Edge effect indicator of protective vegetation mosaic, its value is between 1 and 2. The closer the I_E value is to 1, the more regular and simple the shape of the protective vegetation mosaic tends to be. The larger the I_E value, the more complex the overall shape of the protective vegetation mosaic, the stronger the edge effect, and the stronger its integrity;

P_i—The perimeter of the ith protective vegetation mosaic;

lg—the logarithm with base 10;

100%—Conversion constant, multiplied by Convert the value range of VPI to between 0% and 100%.

(3) Vegetation Quality Integrity (VQI) [2]

The forest vegetation quality assessment takes the areas covered by vegetation in the second-class survey data of forest resources in nature reserves as the research object. The tree age group, crown density, mean diameter at breast height and mean canopy height were classified into different grades, and the vegetation quality indicator of each forest class was calculated. The grade classification and assignment of the participating indicators are shown in Table S1.

Table S1. Grading of indicators for the overall forest vegetation quality indicator

**Evaluation indicators**	**Rating of participating indicators（D_F）**				
	**Ⅰ（Assign a value of 1.00）**	**Ⅱ（Assign a value of 0.62 ）**	**Ⅲ（Assign a value of 0.38）**	**Ⅳ（Assign a value of 0.14）**	**Ⅵ（Assign a value of 0）**
age groups	Overmature and mature natural forest	Near-mature natural forest	Middle-aged and young natural forests	Native plantation	Exotic plantation
crown density	0.7~1.0	0.50~0.7	0.30~0.50	0.20~0.3	0 ~0.2
mean diameter	>25cm	20~25 cm	15~20 cm	10~15 cm	0~10 cm
mean canopy height	>20m	15~20 m	10~15 m	5~10 m	0~5 m

The forest vegetation indicator calculation formula of the forest class is as follows:

$$I_{F_i} = \frac{1}{4}\sum_{d=1}^{4} I_{F_{id}} \quad \text{(S5)}$$

Where:

I_{F_i}—The vegetation quality indicator of the forest sub-compartment, that is, the vegetation quality indicator of the i-th forest sub-compartment, is between 0 and 1;

$I_{F_{id}}$— The grade assignment of the d-th participating indicator of the i-th forest sub-compartment.

The quality indicator of forest vegetation types (VQI) was calculated based on the quality indicator of forest subgroups. The calculation formula is as follows:

$$VQI = \frac{\sum_{i=1}^{n} S_{F_i} \cdot I_{F_i}}{S_F} \times 100\% \quad \text{(S6)}$$

Where:

VQI —The overall quality indicator of forest vegetation, ranging from 0 to 100%;

S_{F_i}—The area of the i-th forest sub-compartment;

n —The number of forest sub-compartment;

100%—Conversion constant, multiplied by To convert the value range of VQI to between 0% and 100%.

S1.2 Forest Ecosystem authenticity

(1) Vegetation Pattern Authenticity (VPA) [1]

$$VPA = \frac{\sum_{i=1}^{n} S_i}{S_V} \times 100\% \quad \text{(S7)}$$

Where:

VPA—The proportion of natural vegetation area, ranging from 0 to 100%;

S_i—The area of the i-th natural vegetation within the nature reserve;

S_V—The total area covered by vegetation within the nature reserve;

100%—Conversion constant, multiplied by it to convert the value range of VPA to between 0 and 100%.

(2) Community Succession Authenticity (CSA) [1]

$$CSA = \frac{\sum_{i=1}^{n} S_{ci}}{S_V} \times 100\% \quad \text{(S8)}$$

Where:

CSA—The proportion of the climax community area, ranging from 0 to 100%;

S_{ci}—The area of the i-th climax plant community within the nature reserve;

S_V—The total area of natural vegetation within the nature reserve;

100%—Conversion constant, multiplied by it to convert the value range of CSA to between 0 and 100%.

(3) Forest Quality Authenticity (FQA) [3]

Evaluation indicators for forest naturalness encompass forest forest stand origin, age groups, soil depth, interference degree, , and forest stand growth status. Each of these indicators is graded, and the naturalness indicator for each forest class is calculated accordingly. The grading divisions and value assignments for the participating indicators are presented in Table S2.

Table S2. Grading of indicators for the Forest Quality Authenticity

**Evaluation indicators**	**Rating of participating indicators**		
	Ⅰ（Assign a value of 1.00）	Ⅱ（Assign a value of 0.62）	Ⅲ（Assign a value of 0.38）
stand origin	natural	Artificial promotion of natural regeneration	artificial
age groups	Overmature and mature forest	Near-mature forest	Middle-aged and young forest
soil depth	>60cm thick	30~59cm medium	<30cm thin
interference degree	slight	moderate	serious
natural degree	1	2	3

Calculation formula of forest naturalness in forest sub-compartment As follows:

$$P_{N_i} = \frac{1}{4}\sum_{d=1}^{4} P_{N_{id}} \tag{S9}$$

Where:

P_{N_i}—Forest sub-compartment naturalness indicator, that is, the naturalness indicator of the i-th forest, is between 0 and 1.

$P_{N_{id}}$—The grade assignment of the d-th evaluation indicator of the i-th forest sub-compartment.

According to the sub-compartment forest naturalness evaluation value (N_i), the area weighted average method was used to construct a comprehensive forest naturalness evaluation indicator. The calculation formula is as follows:

$$FQA = \frac{\sum_{i=1}^{n} S_{N_i} \cdot I_{N_i}}{S_N} \times 100\% \tag{S10}$$

Where:

FQA —The forest naturalness indicator, ranging from 0 to 100%;

S_{N_i}—The area of the i-th forest class;

S_N—The total area of the protected area;

n —The number of small forest sub-compartment;

100%—Conversion constant, multiplied by it to convert the value range of FQA to between 0 and 100%.

S1.3 composite indicator

(1) Forest Ecosystem Integrity (FEI)

The Forest Ecosystem Integrity (FEI) is used to represent the overall integrity of the natural ecosystem within the study area. The calculation formula is as follows:

$$FEI = \sum_{i\in(1,3)}^{n} I_i W_i \tag{S11}$$

Where:

FEI—Forest ecosystem integrity indicator, expressed as a percentage, ranging from 0 to 100%.

n—The number of indicators selected to be suitable for evaluating a certain study area.

I_i—The evaluation value of the selected indicator.

W_i—The weight of the selected indicator, 0<W_i≤1, the sum of the weights of the selected indicators is 1.

(2) Forest Ecosystem Authenticity (FEA)

The Forest Ecosystem Authenticity (FEA) is used to express the authenticity of the natural ecosystem within the study area. The calculation formula is as follows:

$$FEA = \sum_{i\in(1,3)}^{n} P_i W_i \tag{S12}$$

Where:

FEA—Forest ecosystem authenticity indicator, expressed as a percentage, ranging from 0 to 100%。

n—The number of indicators selected to be suitable for evaluating a certain study area.

P_i—The evaluation value of the selected indicator i.

W_i—The weight of the selected indicator, 0<W_i≤1, the sum of the weights of the selected indicators is 1.

S1.3 composite indicator

(1) Forest Ecosystem Integrity (FEI) [1]

The Forest Ecosystem Integrity (FEI) is used to represent the overall integrity of the natural ecosystem within the study area. The calculation formula is as follows:

$$FEI = \sum_{i\in(1,3)}^{n} I_i W_i \tag{S11}$$

In the formula:

FEI——Forest ecosystem integrity indicator, expressed as a percentage, ranging from 0 to 100%.

n——The number of indicators selected to be suitable for evaluating a certain study area.

I_i——The evaluation value of the selected indicator.

W_i——The weight of the selected indicator, 0<W_i≤1, the sum of the weights of the selected indicators is 1.

(2) Forest Ecosystem Authenticity (FEA) [1]

The Forest Ecosystem Authenticity (P_{AU}) is used to express the authenticity of the natural ecosystem within the study area. The calculation formula is as follows:

$$FEA = \sum_{i\in(1,3)}^{n} P_i W_i \tag{S12}$$

In the formula:

FEA——Forest ecosystem authenticity indicator, expressed as a percentage, ranging from 0 to 100%。

n——The number of indicators selected to be suitable for evaluating a certain study area.

P_i——The evaluation value of the selected indicator i.

W_i——The weight of the selected indicator, 0<W_i≤1, the sum of the weights of the selected

indicators is 1.

References

1. Peng, Y.; Huang, Z.; Lin, L.; Wang, R.; Cui, G. Exploring Evaluation Methods for Integrity and Authenticity of Terrestrial Natural Ecosystems in National Parks: The Case of Qianjiangyuan National Park System Pilot. *Biodiversity Science* **2021**, *29*, 1295–1307, doi:10.17520/biods.2021263.
2. Cui, G.; Zhang, J.; Liu, F.; Li, Z.; Guo, H.; Jia, H.; Guo, Z.; Tu, L. *Technical Guidelines for Assessing Conservation Efficiency of Nature Reserves—Part 2: Vegetation Conservation*; **2014**.
3. Chen, Y. Research on the Effect of Forest Protection in Khan Ma NationalNature Reserve ofDaxing’anling Mountains of Inner Mongolia, Beijing Forestry University, 2014.
